# Antibiotic susceptibility pattern among children admitted to a hospital in Nigeria: A retrospective study

**DOI:** 10.4102/ajlm.v13i1.2362

**Published:** 2024-08-20

**Authors:** Aderonke O. Oluwo, Mary A. Lawal, Cecilia A. Mabogunje, Olubunmi T. Okurame

**Affiliations:** 1 Dental Division, Massey Street Children’s Hospital, Lagos, Nigeria; 2 Medical Division, Massey Street Children’s Hospital, Lagos, Nigeria; 3 Laboratory Unit, Massey Street Children’s Hospital, Lagos, Nigeria

**Keywords:** antibiotic resistance, children, bacteria pathogens, susceptibility pattern, antibiotic therapy

## Abstract

**Background:**

The impact of antimicrobial resistance on children living in resource-limited countries has been underreported, despite its established global threat.

**Objective:**

This retrospective study aimed to describe the trend of antibiotic susceptibility in the paediatric age group.

**Methods:**

Sensitivity test report data consisting of 300 paediatric patients aged 18 hours to 192 months were retrieved from the microbiology laboratory records at a state-owned children’s hospital in Nigeria over a period of 4 months starting from December 2021 to March 2022. Five genera (*Escherichia coli, Klebsiella* spp., *Pseudomonas* spp., *Staphylococcus aureus* and *Streptococcus* spp.) were cultured as recommended by the Clinical Laboratory Standard Institute, using the Kirby Bauer disc diffusion method. Antimicrobial susceptibility testing was carried out on isolates using 15 different antibiotics.

**Results:**

*Staphylococcus aureus* was the most frequent pathogen isolated 32.1% (50/156) and *Pseudomonas* spp. was the least frequent pathogen isolated 7.1% (11/156) in all samples. The isolates with the highest rate of resistance to the tested antibiotics were *S. aureus* 32.1% (50/156), *E. coli* 28.2% (44/156) and *Klebsiella* spp. 20.5% (32/156). Isolates in all age groups were more resistant to ampicillin, amoxicillin + clavulanic acid, cefuroxime and cefepime.

**Conclusion:**

Antibiotic resistance is high, especially the younger Nigerian children. Strict antibiotic protocols should be adhered to especially in the use of empirical antibiotic therapy in hospitals.

**What this study adds:**

Our study reveals a higher trend of antibiotic resistance, especially in younger children. It further shows that the pathogens are most resistant to the most available empirical antibiotics in Nigeria.

## Introduction

Antimicrobial resistance (AMR) is a global public health issue for both epidemiologic and economic reasons.^1^ The World Health Organization (WHO) estimates about 700 000 deaths yearly due to multi-drug resistance, with 200 000 deaths occurring in neonates.^2^ Antimicrobial resistance, described as a global crisis, is causing difficulty in the treatment of common bacterial infections in children and is, in some instances, making treatment impossible.^1,2^ This has led to fatal outcomes as evident in an increase in mortality and morbidity among the paediatric age group.^1,3^

Antibiotics are among the most prescribed drugs, both in the hospital and within the community, in the paediatric age group.^3^ The factors that guide such prescriptions are often invalid, especially in sub-Saharan Africa, where self-medication is high and the use of unmeasured herbal medication is common practice.^4^ The inadequate microbiology laboratories and equipment often justifies the empirical use of antibiotics in children.^5,6,7^ The development of bacterial resistance mechanisms is a normal process, regardless of resistance gene acquisition or gene mutation; however, inappropriate or unregulated use is believed to hasten the process.^1^ Abuse of antibacterial agents in humans provides a selective pressure that promotes and spreads resistant strains.^1^ Unnecessary prescriptions used in the management of viral infections and other non-communicable diseases, coupled with inappropriate dosage, have contributed to an increase in the development of multi-drug resistant bacteria species.^5,6,7^

A lack of equipped laboratories, coupled with non-adherence to infection prevention and control practices, increases the risk of AMR, reduces the quality of care, and increases paediatric morbidity and mortality.^5,8,9,10^ In the West African region, absence of monitoring of licensed pharmaceutical vendors has given way to availability of many first- and second-line antibiotics as over-the-counter medications. This unguided practice, alongside the widespread patronage of drug hawkers, reduces the strength of antimicrobial surveillance within the region. Likewise, the pattern of antibiotic resistance is not well studied in children, despite the high usage of antibiotics in this age group. The African continent has a major setback in accrued data on AMR.^9^ The WHO considers Africa to have a wide gap on recorded prevalence of AMR as compared to most other continents.^11^ This can be attributed to weak national surveillance systems in many African nations. For instance, in a 2023 WHO survey,^12^ the One Health approach created by the organisation was found to be efficient in only eight African countries.^12^ The One Health approach aims to prevent and control AMR by employing stakeholders in different disciplines and sectors to work together through surveillance and shared databases.^12^ An earlier report on Central and East African nations clearly defined the inadequacy in antimicrobial susceptibility testing.^13^ This report also indicated that the AMR pattern in these regions may be more ubiquitous than documented.^13^ Recent efforts to define the map of AMR (especially in the paediatric group) in sub-Saharan Africa have proven difficult because of limited data across West African states, thus affecting the formation of comprehensive national guidelines and protocol.^6,10^

The paucity of knowledge on the trends of AMR in Nigerian children has necessitated this research. This study therefore aims to determine the trend of antibiotic susceptibility of identified pathogens cultured from different clinical specimens in a sample of hospitalised Nigerian paediatric patients.

## Methods

### Ethical considerations

Ethical approval was obtained from the Lagos State University Teaching Hospital Health Research and Ethics Committee (LREC/06/10/2193). The ethical approval was obtained in a written format after submission of a detailed study proposal and before data collection. All data-collecting procedures followed the guidelines of the National Health Research Ethics Committee, Nigeria (www.nhrec.net). As this retrospective study involved data of collected specimens with laboratory codes from routine clinical practices, informed consent was waived by the Lagos State University Teaching Hospital Health Research and Ethics Committee according to the Declaration of Helsinki. Data access authorisation and restriction of sensitive records were duly observed according to the national data protection and privacy regulation. Authorised data records were kept in a locked file cabinet accessible only to the principal investigator.

### Study design and population

This was a retrospective study to describe the pattern of susceptibility to antibacterial agents in children over the period from December 2021 to March 2022. The study population comprised inpatients admitted at the Massey Street Children’s Hospital in Lagos, south-western Nigeria. The hospital is currently the only public children’s hospital in the state. It is located in a densely populated suburb of the state and cares for patients from all over the state and its environs.

### Clinical care and laboratory procedures

Clinical specimens, including blood, cerebrospinal fluid, ear swabs, urine, and stool, were collected from hospitalised patients, according to their disease condition, as part of clinical care by the managing physician, and submitted to the microbiological testing unit. At the study site, cultures including urine, ear, and cerebrospinal fluid followed standard microbiological methods such as morphology on culture media, gram staining, and conventional biochemical testing. Blood culture was performed according to the BD BACTEC^TM^ automated blood culture systems (Becton, Dickinson and Company, Sparks, Maryland, United States). All cultures were cultured aerobically, at 37 °C for 18 h – 24 h, and negative cultures were incubated for up to 5 days for bacteria growth before being reported as negative.

### Antimicrobial susceptibility testing

The in-vitro antibiotic susceptibility of the identified pathogens was performed as recommended by the Clinical Laboratory Standard Institute,^14^ using the Kirby Bauer disc diffusion method on Mueller-Hinton agar (HiMedia Laboratories Pvt, Mumbai, India). Five genera (*Escherichia coli, Klebsiella* spp., *Pseudomonas* spp., *Staphylococcus aureus,* and *Streptococcus* spp.) were isolated and tested for antibiotic susceptibility using 15 antibiotics by disc diffusion method. The following antibiotic agents were tested: ampicillin (10 µg), amoxicillin/clavulanic acid (20/10 µg), cefotaxime (30 µg), cefepime (30 µg), oxacillin (1 µg), azithromycin (2 µg), clindamycin (2 µg), cefuroxime (30 µg), ceftriaxone (30 µg), erythromycin (15 µg), cefoxitin (10 µg), meropenem (10 µg), ciprofloxacin (5 µg), ofloxacin (5 µg), and ampicillin + sulbactam (10 µg). Gram-negative bacteria *E. coli* and *Klebsiella* spp. were screened for extended-spectrum beta-lactamase production using ceftazidime (30 µg). The double-disk synergy test for ceftazidime (30 µg), and co-amoxicillin/clavulanic acid (20/10 µg) was used to confirm extended-spectrum beta-lactamase production. Methicillin-resistant *S. aureus* was detected using the cefoxitin (30 µg) disc diffusion test. The susceptibility interpretation (susceptible, intermediate, resistant) of zone clearance was according to the Clinical and Laboratory Standards Institute guidelines.^14^ The American Type Culture Collection *E. coli* 25922 and *S. aureus* 25923 were used as quality control strains for all laboratory methods. Isolates with intermediate or resistant results on antibiotic susceptibility were classified as resistant strains during data analysis.

### Data collection

We extracted data from case record files and microbiology laboratory medical records for 300 paediatric patients aged 18 hours to 14 years, who were admitted and tested in the laboratory unit of the hospital during the study dates. Information on patients’ socio-demographic characteristics, including age and gender, was obtained from the patient’s medical records. Patients with missing registration numbers, incomplete medical records, and those attending the highly active antiretroviral therapy clinic were excluded from the study. Antimicrobial sensitivity test report data were collected from the microbiology records of the laboratory unit of the hospital. Bacterial profiles were obtained by reviewing the laboratory culture database.

### Data analysis

We entered the participants’ demographic characteristics, pathogen isolated, and susceptibility pattern of all isolates into a Microsoft Excel spreadsheet (v.16.0, Redmond, Washington, United States). Participants were divided into 5 subgroups according to their paediatric age: neonate (0–1 month), infant (˃ 1 month – 12 months), preschooler (˃ 12 months – 60 months), school age (˃ 60 months – 120 months), adolescent (˃ 120 months – 192 months). Data were analysed using IBM^®^ SPSS^®^ version 29 (Armonk, New York, United States). The variables (socio-demographic and clinical characteristics, bacterial isolates, and antibiotic susceptibility) were expressed as frequencies, percentages, mean ± s.d., and ratios, as considered applicable.

## Results

A total of 300 participants were enrolled in this study (Table 1). There were 169 (56.4%) boys and 131 (43.6%) girls, with a ratio of 1.3:1. The mean age of the participants was 41.13 ± 52.69 months (range 0–192 months) with the largest number in the neonatal group, and the smallest number in the adolescent group. Blood (51.7%) and urine (30.3%) cultures accounted for the most requested microbiological test. Of the 300 culture results, 156 (52.0%) were culture positive while 144 (48.0%) were culture negative. The highest percentage of positive samples was found in the neonatal group (34.6%). Overall, children in the younger age groups, including neonates (18.0%), infants (10.7%), and preschool children (11.3%) accounted for most of the positive samples (Table 1).

**TABLE 1:** Socio-demographic and clinical data of subjects in Lagos, south-western Nigeria, from December 2021 to March 2022.

Variable	Neonate		Infant		Preschool		School age		Adolescent		Total	
	*n*	%	*n*	%	*n*	%	*n*	%	*n*	%	*n*	%
**Gender**												
Male	50	16.7	36	12.0	38	12.7	35	11.7	10	3.3	169	56.4
Female	41	13.7	25	8.3	28	9.3	28	9.3	9	3.0	131	43.6
Total	91	30.4	61	20.3	66	22.0	63	21.0	19	6.3	300	100.0
**Test culture**												
Blood	77	25.7	31	10.3	26	8.7	17	5.7	4	1.3	155	51.7
Urine	6	2.0	19	6.3	23	7.7	31	10.3	12	4.0	91	30.3
Stool	1	0.3	8	2.7	5	1.7	7	2.3	2	0.7	23	7.7
Ear swab	1	0.3	0	0.0	9	3.0	6	2.0	1	0.3	17	5.7
Cerebrospinal fluid	6	2.0	2	0.7	2	0.7	2	0.6	0	0.0	12	4.0
Wound swab	0	0.0	1	0.3	0	0.0	0	0.0	0	0.0	1	0.3
Eye swab	0	0.0	0	0.0	1	0.3	0	0.0	0	0.0	19	0.3
**Culture specimen**												
Culture negative	37	12.3	29	9.7	32	10.7	37	12.3	9	3.0	144	48.0
Culture positive	54	18.0	32	10.7	34	11.3	26	8.7	10	3.3	156	52.0

Note: Age in months. Neonate (0–1 month), infant (˃ 1 month – 12 months), preschooler (˃ 12 months – 60 months), school age (˃ 60 months – 120 months), adolescent (˃ 120 months –192 months). Mean age = 41.1 ± 52.69 months.

### Age distribution among culture-positive samples

Of the 156 (52.0%) positive samples, boys had a higher percentage of positive samples in the neonate (37.8%), preschool (23.2%), and adolescent (11.5%) groups; the infant (23.0%) and school-age (18.9%) groups recorded a slightly higher percentage of positive samples among girls (Table 2). *Staphylococcus aureus* was the most frequent organism isolated (32.1%), and *Pseudomonas* spp. was the least frequent organism isolated (7.1%) among all samples (Table 2). *Staphylococcus aureus* was the most frequently isolated organism in the neonatal (13.5%) and adolescent (3.2%) groups, whereas *E. coli* was the most isolated organism in all the age groups except for the neonatal (7.1%) adolescent (0.6%) age groups. *Pseudomonas* spp. was not isolated among infants and was infrequently detected in the neonatal and adolescent groups (both 0.6%). *Streptococcus* spp. was not detected in the adolescent group as compared to the younger age groups.

**TABLE 2:** Age distribution among culture positive samples and in relation to gender, Lagos, Nigeria, from December 2021 to March 2022.

Pathogen	Neonate		Infant		Preschool		School age		Adolescent		Total	
	*n*	%	*n*	%	*n*	%	*n*	%	*n*	%	*n*	%
**Positive culture**												
Male	31	37.8	15	18.3	19	23.2	12	14.6	5	11.5	82	52.7
Female	23	31.1	17	23.0	15	20.3	14	18.9	5	8.8	74	47.3
**Isolated pathogens**												
*Escherichia coli*	11	7.1	13	8.3	11	7.1	8	5.1	1	0.6	44	28.2
*Klebsiella* spp.	10	6.4	9	5.8	5	3.2	5	3.2	3	1.9	32	20.5
*Pseudomonas* spp.	1	0.6	0	0.0	6	3.9	3	1.9	1	0.6	11	7.1
*Staphylococcus aureus*	21	13.5	8	5.1	9	5.8	7	4.5	5	3.2	50	32.1
*Streptococcus* spp.	11	7.0	2	1.3	3	1.9	3	1.9	0	0.0	19	12.1

**Total**	54	34.6	32	20.5	34	21.9	26	16.7	10	6.3	156	100.0

Note: Age in months. Neonate (0–1 month), infant (˃ 1 month – 12 months), preschooler (˃ 12 months – 60 months), school age (˃ 60 months – 120 months), adolescent (˃ 120 months – 192 months).

### Pattern of drug resistance among detected isolates

The most frequently recovered isolates, including *S. aureus* (*n* = 50), *E. coli* (*n* = 44) and *Klebsiella* spp. (*n* = 32), were also the most often resistant to the tested antibiotics (Table 3). Isolates were most often resistant to ampicillin (*n* = 58), amoxicillin + clavulanic acid (*n* = 54), and cefepime (*n* = 53), compared to the other antibiotics tested.

**TABLE 3:** Pattern of drug resistance in culture positive samples of children in Lagos, south-western Nigeria, from December 2021 to March 2022.

Isolates	Isolated pathogens					
	*Escherichia coli*	*Klebsiella* spp.	*Pseudomonas* spp.	*Staphylococcus aureus*	*Streptococcus* spp.	Total
**Number of isolates**	44	32	11	50	19	156
**Antibiotic *(number of resistant isolates)***						
Ampicillin	18	16	7	13	4	58
Amoxicillin + clavulanic acid	9	9	6	19	11	54
Cefotaxime	7	3	0	9	5	24
Cefepime	15	9	4	22	3	53
Oxacillin	1	0	0	1	0	2
Azithromycin	10	5	1	14	1	31
Clindamycin	0	0	0	1	0	1
Cefuroxime	10	0	0	1	0	11
Ceftriaxone	5	4	0	14	2	25
Erythromycin	1	1	0	4	2	8
Cefoxitin	2	0	0	1	0	3
Meropenem	4	1	0	2	2	9
Ciprofloxacin	0	0	0	1	1	2
Ofloxacin	0	1	0	3	0	4
Ampicillin + sulbactam	2	0	0	5	2	9

### Antibiotic susceptibility pattern among different age groups

Antibiotic susceptibility patterns varied by age group and isolate (Table 4). Younger age groups were more resistant to the antibiotics tested as compared to isolates from the older school-age and adolescent groups. Isolates were resistant to 11 of 15 drugs tested in the neonatal group in contrast to isolates from the school-age group, which were more sensitive to 10 of 15 drugs tested. Among all the drugs tested, isolates demonstrated more resistance to ampicillin (neonate 73.7%, infant 88.7%, preschool 82.4%, school age 85.7%) and cefuroxime (neonate 56.7%, infant 73.3%, preschool 52.4%). In contrast, isolates showed more sensitivity to meropenem (neonate 84.6%, infant 88.2%, preschool 84.2%, school age 85.7%, adolescent 100.0%) and erythromycin (neonate 81.2%, infant 100.0%, preschool 66.7%, school age 100.0%, adolescent 75.0%) in the majority of the age groups. Isolates from the preschool group were more resistant to amoxicillin + clavulanic acid (58.3%), cefotaxime (54.5%), and cefuroxime (52.4%). This differed from the infant group which exhibited more resistance pattern to cefepime (52.0%), azithromycin (66.7%), and cefuroxime (73.3%). Likewise, isolates detected in the neonatal group were more resistant to ceftriaxone (57.1%) and ofloxacin (66.7%), as compared to the rest of the subgroups.

**TABLE 4:** Susceptibility pattern from positive samples among different age groups from Lagos, south-western Nigeria, from December 2021 to March 2022.

Antibiotics	Susceptibility	Neonates		Infants		Preschool		School		Adolescents	
		*n*	%	*n*	%	*n*	%	*n*	%	*n*	%
Ampicillin	S	5	26.3	2	11.3	3	17.6	2	14.3	3	60.0
	R	14	73.7	16	88.7	14	82.4	12	85.7	2	40.0
Amoxillin + clavulanic acid	S	24	54.5	16	66.7	10	41.7	10	62.5	3	37.5
	R	20	45.5	8	33.3	14	58.3	6	37.5	5	62.5
Cefotaxime	S	13	59.1	3	33.3	5	45.5	6	75.0	4	66.7
	R	9	40.9	6	66.7	6	54.5	2	25.0	2	33.3
Cefepime	S	24	57.1	12	48.0	15	53.6	12	63.2	6	66.7
	R	18	42.9	13	52.0	13	46.4	7	36.8	3	33.3
Oxacillin	S	4	100.0	2	100.0	0	0.0	-	-	1	100.0
	R	0	0.0	0	0.0	2	100.0	-	-	0	0.0
Azithromycin	S	13	100.0	4	33.3	10	58.8	9	90.0	4	66.7
	R	0	0.0	8	66.7	7	41.2	1	10.0	2	33.3
Clindamycin	S	1	100.0	-	-	1	100.0	-	-	0	0.0
	R	0	0.0	-	-	0	0.0	-	-	1	100.0
Cefuroxime	S	13	43.3	4	26.7	10	47.6	9	56.2	4	50.0
	R	17	56.7	11	73.3	11	52.4	7	43.8	4	50.0
Ceftriaxone	S	9	42.9	3	60.0	9	64.3	6	66.7	3	60.0
	R	12	57.1	2	40.0	5	35.7	3	33.3	2	40.0
Erythromycin	S	13	81.2	4	100.0	8	66.7	8	100.0	3	75.0
	R	3	18.8	0	0.0	4	33.3	0	0.0	1	25.0
Cefoxitin	S	-	-	1	100.0	2	66.7	1	33.3	1	100.0
	R	-	-	0	0.0	1	33.3	2	66.7	0	0.0
Meropenem	S	11	84.6	15	88.2	16	84.2	12	85.7	5	100.0
	R	2	15.4	2	11.8	3	15.8	2	14.3	0	0.0
Ciprofloxacin	S	8	80.0	5	100.0	5	100.0	3	100.0	2	100.0
	R	2	20.0	0	0.0	0	0.0	0	0.0	0	0.0
Ofloxacin	S	1	33.3	0	0.0	-	-	-	-	-	-
	R	2	66.7	2	100.0	-	-	-	-	-	-
Ampicillin + sulbactam	S	5	83.3	3	50.0	3	75.0	3	100.0	-	-
	R	1	16.7	3	50.0	1	25.0	0	0.0	-	-

Note: Age in months; Neonate (0–1 month), infant (˃ 1 month – 12 months), preschooler (˃ 12 months – 60 months), school age (˃ 60 months – 120 months), adolescent (˃ 120 months –192 months).

S, sensitive; R, resistant.

## Discussion

This retrospective study demonstrated the varying pattern of antibiotic susceptibility in children. The bacterial species studied have shown strong associations with drug resistance globally and are part of the most important consideration of the WHO’s research for new antimicrobial discovery and development.^15^

Our results show that the highest number of positive samples were from the neonatal group, and blood specimens contributed to more than half of specimens processed. This observation probably points to the alarming rate of neonatal sepsis and the rising cases of septicaemia among children, especially in sub-Saharan Africa. Our findings are in agreement with previous reports from Nigeria and Gambia.^16,17^

The pathogenic organisms studied are part of the list of emerging bacteria threats as highlighted by Fair et al.^18^ We agree with this report, as our findings revealed similar trends, with *S. aureus, E. coli* and *Klebsiella* spp. having the highest resistance profile among the drugs tested.^18^

*Staphylococcus aureus*, which was the most isolated organism among all the subjects studied, also had the highest resistance profile. Cassat et al.^19^ in their review emphasised the rapidly evolving nature of *S. aureus*, producing aggressive resistant strains.^19^ This organism is also believed to be the most prevalent infective paediatric pathogen in several parts of the world, posing a significant threat without newer therapeutic options.^18,19^

The isolates included in this study also exhibited more resistance to ampicillin, amoxicillin + clavulanic acid, cefuroxime and cefepime. Previous African studies have reported high resistance to all the drugs except for cefepime.^20,21,22,23^ The high prevalence observed in our study may be explained by the fact that information obtained were from records of patients on admission who were more likely to require antimicrobials compared with outpatients. Likewise, the observed high rate of resistance could be associated with pre-exposure to a wide variety of antibiotics with optimal or sub-optimal dosages before their referral to our hospital. We also note that ampicillin, amoxicillin + clavulanic acid, cefuroxime and cefepime are all under the WHO Access, Watch, Reserve classification of antibiotics to support antimicrobial stewardship programmes in the hospitals.^24^ These drugs, recommended by the WHO, are the most prescribed to children currently by physicians, especially in suburban and rural Nigeria. Resistance to these drugs suggests a disturbing shift to antibiotics considered reserved under the WHO classification intended for severe infections.^24,25^

We recorded a higher resistance pattern in younger children compared with school aged children and adolescents. This is similar to previous documented reports from different parts of Africa.^3,10,17,26,27,28,29^ Children, especially those under 5 years of age, receive more antibiotics than adults due to frequent bouts of infections. Furthermore, blurring insights to mechanism of paediatric pathogenic organism, coupled with scarce data on resistance patterns in this age group, may increase antibiotic resistance in these age groups.^30^ This may be especially true in sub-Saharan Africa, which is considered a resource-poor region.

This study also noted the high resistance profile of isolates within the neonatal group. This observation is in resonance with previous reports on the mounting resistance pattern in neonatal sepsis.^31,32^ These authors have also noted that *E. coli* and *Klebsiella* spp. are dominant resistant pathogens isolated in neonatal sepsis. From our results, meropenem remains an available option as it shows a high sensitivity rate; however, this drug has been designated by WHO as a reserved drug and should be used under strict antibiotic policy.^24^

### Limitations

The data used in this retrospective study were from records of hospitalised children in our facility who may have been infected with multi-drug resistant bacterial pathogens not reported in our study. The presence of additional multi-drug resistant pathogens not included in our analyses could have altered the susceptibility pattern reported here. However, our findings can be used as part of ongoing arguments on national awareness programmes on antimicrobial resistance in Nigeria.

### Conclusion

Our study demonstrated a higher antibiotic resistance in younger children compared with school aged children and adolescents. The pathogens studied were resistant in all age groups to the most available and empirically used antibacterial agents in Nigeria: ampicillin, amoxicillin + clavulanic acid, cefuroxime, and cefepime. In awareness of this emerging threat, we believe this is a stimulation towards strengthening antimicrobial stewardship programmes and effective surveillance systems following current WHO guidelines at local, state and national levels in Nigeria.
